# Overexpressing *PpBURP2* in Rice Increases Plant Defense to Abiotic Stress and Bacterial Leaf Blight

**DOI:** 10.3389/fpls.2022.812279

**Published:** 2022-05-06

**Authors:** Shunwu Yu, Fangwen Yang, Yuqiao Zou, Yunan Yang, Tianfei Li, Shoujun Chen, Yulan Wang, Kai Xu, Hui Xia, Lijun Luo

**Affiliations:** Shanghai Agrobiological Gene Center, Shanghai Academy of Agricultural Sciences, Shanghai, China

**Keywords:** BURP protein, abiotic stress, moss, rice, gene evolution

## Abstract

Mosses are one of the earliest diverging land plants that adapted to living on land. The BURP domain-containing proteins (BURP proteins) are plant-specific proteins that appeared when plants shifted from aquatic environments to land. Phylogenetic analysis revealed that the BURP domain of higher plants is originated from lower land plants and divergent because of motif conversion. To discover the function of BURP protein in moss, rice transgenics with ectopic expression of *PpBURP2* were subjected to different abiotic stresses treatments. The results revealed that the ectopic expression of *PpBURP2* enhanced the tolerance to osmotic and saline stresses at the seedling stage and drought stress at the adult stage. Further ectopic expression of *PpBURP2* improved the cadmium (2+) (Cd^2+^) tolerance and reduced Cd^2+^ accumulation in rice leaves. Transcriptomic analysis of the transgenic *PpBURP2* plants showed that the differentially expressed genes were involved in the metabolism of secondary metabolites, energy, oxidation-reduction process, and defense-related genes. Further experiments showed that the photosynthetic efficiency and resistance against bacterial leaf blight were obviously improved in transgenic plants. Yeast two-hybrid and bimolecular fluorescence complementation (BiFC) assays revealed the physical interaction of BURP domain protein from rice and moss with mitogen-activated protein kinase kinase (MKK) from rice. Therefore, our findings demonstrate that overexpressing *PpBURP2* in rice confers resistance to abiotic stresses and bacterial leaf blight. They also suggested that the regulatory role of BURP-like proteins across lower and higher plants was evolutionary conservation of responses of different classes of plants to different environmental challenges.

## Introduction

The terrestrial environment often incurs extreme variations in water and chemical availability and temperature, as well as increased exposure to radiation compared to the aquatic environment. Adaptation impelled plant morphological and physiological evolution to the conquest of land. Mosses are one of the earliest plants to become adapted to living on dry land and are also one of the largest groups of land plants that cover the world in a variety of habitats. Thus, moss is thought to occupy an ideal phylogenetic position for reconstructing ancient evolutionary changes in genomes that occurred over time (Rensing et al., 2008; Zhang et al., 2020). Plant-specific proteins appeared during the process of the conquest. The function genomics research of plant-specific protein in moss will provide a better understanding of land plant evolution.

The BURP domain-containing protein (BURP protein) is a plant-specific protein that contains a conserved BURP domain at the C-terminus. The amino acid sequence motif of the domain was initially identified in brassicar napus microspore-drived embryo (BNM2) (Treacy et al., 1997), uknown seed protein (USP) (Fiedler et al., 1993), responsive to dehydration 22 (RD22) (Yamaguchi-Shinozaki and Shinozaki, 1993), and beta subunit of polygalacturonase isoenzyme 1 (PG1β) (Moore and Bennett, 1994), and is designated as the BURP domain (Hattori et al., 1998). Later studies found that the proteins are widely present in plants. The completion of genome sequencing of many species is more conducive to the discovery of genes. Genome-wide identification of BURP domain-containing genes was performed in rice, soybean, maize, sorghum, poplar, *Medicago truncatula*, and cotton (Ding et al., 2009; Xu et al., 2010; Gan et al., 2011; Shao et al., 2011; Li et al., 2016; Sun et al., 2019), whereafter, expression analysis in these species revealed that BURP domain-containing genes are closely related to plant development and stress response.

Based on phylogenetic analysis of the putative BURP proteins, the BURP family members in rice can be classified into four or seven subfamilies (Ding et al., 2009; Li et al., 2016). However, the BURP family in Arabidopsis only includes five members, and they are divided into three subfamilies, namely, BNM2-like, RD22-like, and PG1b-like (Ding et al., 2009). AtUSPL1, a homolog of BnBNM2 and VfUSP, is associated with seed development and is deposited in protein storage vacuoles in Arabidopsis (Van Son et al., 2009). PG1b-like protein in Arabidopsis was found to be localized to the cell wall and promotes cell enlargement (Park et al., 2015). In other species, OsBURP16, a PG1b-like protein, was thought to decrease pectin content and cell adhesion and increase abiotic stress sensitivity in rice (Liu et al., 2014). SALI3-2, a vacuole-localized USP-like protein, was found to enhance tolerance to cadmium and copper stresses in soybean (Tang et al., 2014). The function of RD22-like proteins is described in many species. GmRD22 was found to be localized to apoplast and enhance tolerance toward abiotic stress in soybean (Wang et al., 2012). Analysis of expression pattern and transgenic plants revealed that the RD22-like gene is associated with abiotic stresses (Yu et al., 2004; Ding et al., 2009; Xu et al., 2010; Li et al., 2016; Dinh and Kang, 2017; Phillips and Ludidi, 2017). However, RD22-like protein is also found to be associated with plant development. For example, GhRDL1 is localized in the cell wall and interacts with GhEXPA1 to loosen the cell wall and increase seed size in cotton (Xu et al., 2013). Therefore, BURP proteins may be important for plant development and environmental adaptation in plant evolution.

The absence of BURP proteins in hydrobiotic green algae and its presence in non-vascular mosses suggest that they appear when plants shifted from aquatic to land environments (Wang et al., 2015). However, its function remains unclear, especially in lower plants. In this study, PpBURP2 was isolated from moss and its ectopic expression in rice was found to improve the resistance to a variety of abiotic and biotic stresses.

## Materials and Methods

### Plant Material and Growth Conditions

Japonica rice (*Oryza sativa* L. var Nipponbare) was used in this study. Moss (*Physcomitrella patens* subsp. Patens) was isolated from a lawn under an osmanthus tree. Rice seeds were germinated at 30°C for 2 days and transplanted to the growth chamber at 60% relative humidity with 16 h white light (50 μmol/m^2^/s) at 30°C and 8 h dark at 26°C. At the four-leaf stage, the seedlings were treated with 18% (m/v) polyethylene glycol 6,000 (PEG6000), 150 mM NaCl, and 750 mM CdCl_2_ at seedling stage. Rice seedlings were transplanted into 1.5 m tall plastic vessels for drought stress and 30 cm tall plastic vessels for CdCl_2_ stress at the adult stage.

### Identification of BURP Domain-Containing Genes

BURP domain-containing genes were collected from four fully sequenced genomes representing four plant lineages, such as multicellular green algae moss^1^, *Selaginella moellendorffii*^2^, monocotyledonous angiosperms rice^3^, and dicotyledonous angiosperms *Arabidopsis thaliana*^4^. In the study, some identified BURP genes have more than one alternative splicing product. The gene members that could translate the longest protein were selected for further analysis.

### Sequence Analysis of BURP Family Genes

The multiple alignments of protein sequences of BURP domain-containing genes were performed using ClustalX 2.1 for phylogenetic analysis (Larkin et al., 2007). The profiles of the created alignment protein sequences were used to construct a maximum-likelihood phylogenetic tree by the Molecular Evolutional Genetics Analysis version 5 (MEGA5.0) (Tamura et al., 2007). To identify the conserved motifs in BURP proteins, online MEME was employed to analyze the protein sequences of BURP members with the following parameters: maximum number of motifs = 20 (Bailey et al., 2009). The signal peptide sequence was predicted using the online SignalP 4.1 Server (Nielsen, 2017). The repeat architecture was identified in RADAR^5^ (Heger and Holm, 2000). Protein-protein interactions were predicted using the online RicePPINet Server (Liu et al., 2017). Homologs of proteins were analyzed using blastp in Animalia (taxid:33208), Bryophyta (taxid:3208), Chlorophyta (taxid:3041), and charophytes (taxid:3146) organisms on the NCBI database with expect threshold 0.05^6^.

### Cloning of the Full-Length cDNA

Total RNA was used to synthesize first-strand cDNA through the avian myeloblastosis virus (AMV) reverse transcript kit (Transgen, China). A degenerate sense primer, B01 (5′-TGCCACAABDTKRTBTTCCC-3′), and anti-sense primer, B02 (5′- TCRTTCTCVGCNGWCCAATGACA-3′), were designed according to the conserved polypeptide sequences in the BURP domain and used for the PCR amplification of the BURP cDNA. The PCR products were sequenced for specific designed nested primers. For the 5′-rapid amplification of cDNA ends (RACE), first-strand cDNA was synthesized from 5 μg of total RNA, in accordance with the protocol for the 5′ RACE System using adapter primer (AP) (Invitrogen, United States), and was tailed with oligo (dC) according to the manufacturer’s instructions. The first round of PCR was performed with the B02 and AAP primers. Two nested 5′-specific primers, B51 (5′-CTACTGAGGCTGGGCACGCTCTTTCT-3′) and B52 (5′-CTTTCCCTCTTCAACTGAGTGCTTC-3′) were used for two rounds of amplification of the 5′ cDNA ends with the Abridged Universal Amplification (AUAP) primer. To obtain a full-length cDNA from *PpBURPs*, 5′-RACE was performed using RT-PCR based on the conserved region of the BURP gene family. The 5′-RACE assays were performed according to the manufacturer’s instructions with minor modifications. The degenerate primers B01 and B02 were then used to amplify a 203 bp PCR fragment from moss, and the sequences were confirmed by comparison against *PpBURP* genes. Two additional primers were designed based on the confirmed sequence and were used for the isolation of the 5′-ends of the cDNA. These fragments, along with the 203 bp fragment that had been amplified before, were assembled into a 1,198 bp cDNA contig. The PCR products were purified and cloned into pMD18-T vectors for sequencing.

### Molecular Cloning and Transformation of Rice

The full-length cDNA of *PpBURP2* was inserted into the pDONR207 vector (Invitrogen, United States) by the BP recombination reaction. LR reaction between the entry clone and the destination vector pCB4004 produced the expression plasmids pCB4004- *PpBURP2*, in which *PpBURP2* was driven by the CAMV35S promoter. The construct was transformed into the *japonica* rice cv. Zhonghua11 using the agrobacterium-mediated method.

### Reverse Transcription and Quantitative PCR Analysis

Total RNA from different tissues was extracted using TRIzol™ reagent (Invitrogen) after treatments. The first-strand cDNA was synthesized using a PrimeScript RT Reagent Kit with the gDNA Eraser protocol (Transgen, China). Quantitative PCR was performed in a 96-well plate with a Bio-Rad CFX96 Real-Time PCR system (Bio-Rad, Hercules, CA, United States). Reactions were performed with a 20 μl final volume containing 10 μl of SYBR Green Premix Ex Taq (Transgen, China), 1 ng cDNA, and 200 nM gene-specific primers. Relative gene expression was calculated by the 2^–ΔCT^ method, and the *OsActin1* and *PpActin* genes were used as an internal control. All primers used for qPCR are given in Supplementary Table 7.

### Detection of Cd^2+^, Lignin Content, Relative Electrolyte Leakage, and Reactive Oxygen Species

Cadmium (Cd) in leaves and soil was determined by graphite furnace atomic absorption spectrophotometry (Chinese Standard GB/T 17141-1997). After treatment with 150 mM NaCl or 20% PEG6000 for 3 days, leaves of 10-day-old seedlings were collected for physiological analysis. Relative ion leakage was assayed following a previously described method (Liu et al., 2014). Total lignin content was estimated according to previously described methods (Lin and Kao, 2001). Superoxide dismutase (SOD) activity was measured according to the manufacturer’s protocol (Nanjing Jiancheng Bioengineering Institute, China). Three-day-old seedlings after germination were treated with 100 mM NaCl for 1 days and infiltrated with 0.5 mg/ml 3,3′-diaminobenzidine (DAB) as described previously (Fryer et al., 2002).

### Yeast Two-Hybrid Assays

Yeast two-hybrid assays were carried out with a Matchmaker™ Gold Yeast Two-Hybrid System (Clontech, Mountain View, CA, United States). The coding region of *OsMKK1* was cloned into pGBKT7, and the sequences of *OsBURP3* and *PpBURP2* into pGAD-T7. These vectors were subsequently co-transformed into the Y2H Gold strain (Clontech, Mountain View, CA, United States). Protein interactions were evaluated by transformant growth assays on SD/-Leu/-Trp/-His and SD/- Leu/-Trp/-His-/Ade at 30°C for 3–5 days.

### Sub-Cellular Localization and Fluorescence Detection

To investigate the subcellular localization of the PpBURP2 protein, the full-length of PpBURP2 was fused with green fluorescent protein (GFP) into the plant expression vector pCAMBIA1300EGFP after being digested by *Xba*I and *BamH*I. For bimolecular fluorescence complementation (BiFC), the bait gene with the C-terminal portion of YFP (cYFP), and the prey gene with the N-terminal portions of YFP (nYFP) were constructed into the plant expression vector pCAMBIA1300. These vectors were transformed into *A. tumefaciens* strain EHA105, and the transformed agrobacterium were infiltrated into tobacco leaves as described previously (Liu et al., 2010). Two strains with cYFP and nYFP were mixed and infiltrated into tobacco leaves. These agroinfiltrated plants were allowed to grow for 48 h before their fluorescence was examined under a Nikon fluorescence microscope, Japan. For tobacco cells, plasmolysis was performed by incubating the leaves in 20% PEG6000 solution for 3 min.

### RNA Sequencing

Three biological replicates (3 plants each) of wild-type (WT) and PpBURP-OE1 plants were sampled for RNA sequencing. The total RNA was extracted using the TRIZol™ Reagent (Life Technologies). RNA-seq library was constructed with the TruSeq RNA Sample Preparation v2 Guide (Illumina), and RNA sequencing was conducted with Illumina Hiseq 2,500 at Shanghai Personal Biotechnology Co., Ltd. (Shanghai, China). After filtering adapters and low-quality reads, the paired-end reads were then aligned to the reference genome of rice using HISAT2 v2.1.0 (Kim et al., 2019). Fragments per kilobase per million mapped (FPKM) reads was then calculated to estimate the expression level of the genes. Analysis of Gene Ontology (GO)^7^ enrichment was implemented by GOseq in R/Bioconductor packages based on the Wallenius non-central hypergeometric distribution (Young et al., 2010).

### Chlorophyll Fluorescence Imaging

The leaves of the four-leaf stage the seedlings were used for chlorophyll fluorescence measurement at the same time. The chlorophyll fluorescence was determined by using a CF Imager (Technologica, Essex, United Kingdom) as described by Baker (2008). Briefly, values of minimal (Fo) and maximal (Fm) fluorescence were obtained from dark-adapted leaves for 30 min before receiving a saturating light pulse (6,788 μmol photons m^–2^ s^–1^ for 1 s) and lighting (800 μmol photons m^–2^ s^–1^) for 30 min every hour per cycle for 24 cycles. Measurements were performed in at least four leaves from different plants for each treatment.

### Blight Resistance Assay

For examination of resistance to bacterial blight, *Xanthomonas oryzae* pv. oryzae and the mixture of Enshi strain V1 and V2 was prepared following the method of Wang et al. (2003). The uppermost fully expanded leaves of mutants and WT plants were inoculated by the leaf chipping method at the seedling stage (Kauffman et al., 1973).

## Results

### Identification and Analysis of BURP Proteins

We used Protein Blast (BLASTP) to search the genomes of moss (*Physcomitrella patens*) and lycopod (*Selaginella moellendorffii*) with the hidden markov model (HMM) Profile of the BURP domain as a query. Nine putative BURP genes, designated with *PpBURP1* to *PpBURP9*, were identified in moss, while six putative BURP genes, designated with *SmPG1* to *SmPG6*, were identified in lycopod (Supplementary Table 1). Five BURP genes in Arabidopsis were designated according to previous reports (Hattori et al., 1998; Van Son et al., 2009; Harshavardhan et al., 2014) and 17 BURP genes in rice were designated according to Ding’s report (Ding et al., 2009)^8^ Rice Genome Annotation Project Release. Intron–exon organization of all *BURP* genes reveals that introns were apt to insert into 5′-end of gene region, but was rarely inserted into BURP domain (Supplementary Figure 1). This indicates that the organization of the BURP domain is conserved in plants.

**FIGURE 1:**
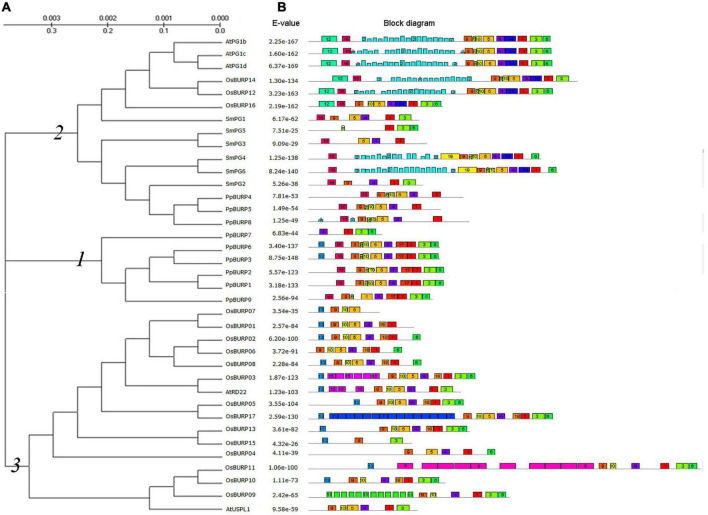
Phylogenetic relationship and motif structures of BURP domain-containing proteins from diverse species. **(A)** The unrooted tree was generated using the Molecular Evolutional Genetics Analysis version 5 (MEGA5) program by the Maximum-likelihood method. The bootstrap values were indicated at the lines. The abbreviations of species names are as follows: At, *Arabidopsis thaliana*; Os, *Oryza sativa*; Pp, *Physcomitrella patens*; Sm, *Selaginella moellendorffii*. **(B)** Multiple expectation maximizations for motif elicitation (MEME) motif search results. Conserved motifs are indicated in numbered, colored boxes. *E*-value is shown on the left. The accession number is shown in Supplementary Table 2.

To uncover the phylogenetic relationships within 38 full-length protein sequences from moss, lycopod, Arabidopsis, and rice, the alignment profiles of protein sequences created by ClustalX2 were used to construct a phylogenetic tree (Figure 1). Based on the domain composition and phylogenetic relationship of the protein sequences, the 38 plant BURP proteins can be split into three main subfamilies (Figure 1). Subfamily 1 is only composed of the proteins with complete BURP domain from moss, and PpBURP4, 5, 7, and 8 proteins with incomplete BURP domain belong to Subfamily 2. Subfamily 2 is mainly composed of PG1β-like proteins, while Subfamily 3 is composed of RD22-like and USP-like proteins. This suggests that BURP proteins originated from Subfamily 1 and differentiated during species evolution.

A typical BURP protein contains four domains: signal peptide, conserved domain, repeat domain, and BURP domain (Granger et al., 2002). A motif online detection, multiple expectation maximizations for motif elicitation (MEME), was used to investigate possible mechanisms of the structural evolution of BURP proteins (Bailey et al., 2009), and 20 conserved motifs were detected (Figure 1 and Supplementary Table 2). In moss, the origin of BURP domain contains motifs 1, 3, 4, 5, 6, 9, and 10, and in the higher plant, the divergent motifs of BURP domain are mainly motif 20 and motif 17, which were lost or substituted. PG1β-like proteins retain motif 20 and motif 17 and convert them into motif 14, but RD22-like proteins lose motif 20 and motif 17 and convert them into motif 18. We found that the original BURP proteins lacked the repeat domain. There were two types of motifs in the conserved domain. Most of the PG-like proteins retained the motif 16, but most of RD22-like and USP-like proteins retained the motif 13 (Figure 1). The analysis of the online SignalP 4.1 Server revealed that the signal peptide was present in a majority of BURP proteins (Supplementary Table 2; Nielsen, 2017). The above results revealed that there were similar divergent evolutions in BURP proteins between monocotyledonous and dicotyledonous plants, but only PG-like proteins occurred in lycopod plants. Therefore, it indicated that BURP proteins in moss are the origin of BURP proteins in higher plants and are related to plant development and stress response.

### Gene Cloning of *PpBURP2*

To know what roles *PpBURP* genes play in the resistance to environmental stress during the move from aquatic to terrestrial environments, the full-length cDNA was cloned using the 5′-RACE method (detail in section “Materials and Methods”) and designated as *PpBURP2* (Gene Bank accession number: JQ290006) containing a complete 1,101 bp coding frame and a 94 bp 5′-untranslated region. The length of the coding frame is longer than the putative *PpBURP2* gene from the moss genome annotation (see text footnote 1) because of 17 amino acid (aa) that was added at the N-terminal of the putative protein. There is a single nucleotide polymorphism in the coding region between *PpBURP2* and putative *PpBURP2.* The cloned PpBURP2 only contains three domains of the BURP family except for the repeat domain (Figure 1).

### Subcellular Localization and Protein Interaction of *PpBURP2*

**FIGURE 2:**
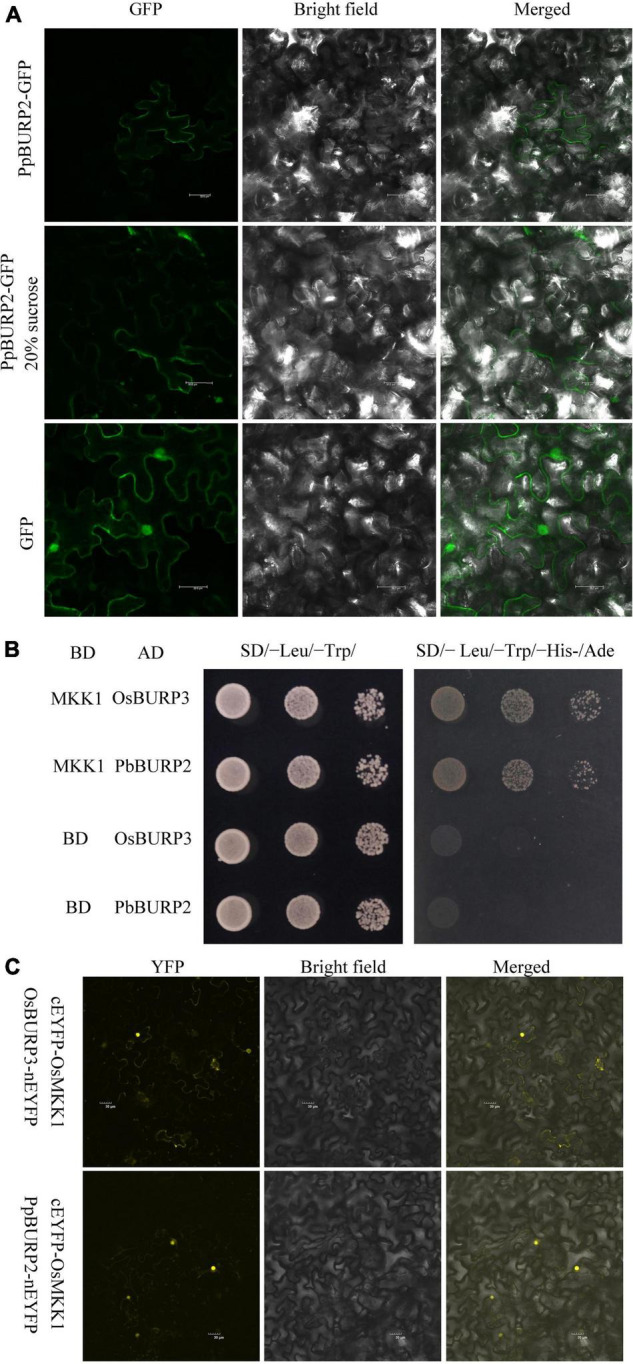
Subcellular Location and protein interaction of PpBURP2. **(A)** Subcellular location green fluorescent protein (GFP) and PpBURP2-GFP fusion gene under the control of the CaMV35S promoter separately and transiently expressed in the tobacco epidermal cells. Leaves of 35S:PpBURP2-GFP tobacco before (top) and after (middle) treatment with 20% sucrose for plasmolysis, as well as 35S::GFP (bottom). The white bar represents 50 μM. **(B)** BURP protein interacting with OsMKK1 using the yeast-two-hybrid method. *OsMKK1* gene was cloned into pGBKT7, and *OsBURP3* gene and *PpBURP2* gene into pGAD-T7, respectively. All of the combinations grew on SD/-Leu/-Trp/, and SD/- Leu/-Trp/-His-/Ade medium at 30°C for 3–5 days. **(C)** Bimolecular fluorescence complementation (BiFC) assays in tobacco leaves indicated that the OsMKK1 interacted with PpBURP2 and OsBURP3 *in vivo*. The *OsMKK1* gene was cloned into cEYFP vector and *OsBURP3*, and *PpBURP2* were cloned into nEYFP vector. Agrobacteria containing the above respective plasmids were transformed into leaves of 5-week-old *N. benthamiana* plants. Scale bar = 30 μM.

To study subcellular localization patterns of PpBURP2, the open reading frame (ORF) was fused in frame with GFP and was expressed in tobacco leaf cells using agrobacterium infection. The distribution of the GFP expression signal in leaf cells revealed that fluorescence of the PpBURP2–GFP fusion protein was depicted in the contors of cells (Figure 2A). After plasmolysis, the signal was present in the cell wall, as well as the space between cell walls (Figure 2A). The GFP signals demonstrate that PpBURP2 protein associates with apoplast.

RicePPINet is a powerful tool to discover new physically interacting proteins in rice (Liu et al., 2017). To find the interaction proteins of BURP domain proteins, OsBURP03 (LOC_Os01g53240) was searched in RicePPINet as a bait protein, and 17 proteins were prioritized with a value over 0.8 (Supplementary Table 3). Surprisingly, there were nine kinase proteins, six of which were the putative receptor-like protein kinases and mitogen-activated protein kinase kinases (MKKs). This indicated that the BURP domain protein is an important unknown member in the signal pathway of the stress response. To confirm the physical interaction, a yeast two-hybrid assay was performed, and the results revealed the interaction among OsMKK1, OsBURP3, and PpBURP2 (Figure 2B). Only the BURP domain is homologous between OsBURP3 and PpBURP2. It can be inferred that OsMKK1 can interact with BURP domain protein from a lower to a higher plant. The interaction between OsMKK1 and OsBURP3 or PpBURP2 was further confirmed by BiFC assays in tobacco leaves (Figure 2C). The fluorescence for both pairs of interacting proteins was observed in the nucleus and cytoplasm (Figure 2C). The results suggested that BURP proteins from lower to higher plants could interact with OsMKK1 in eukaryotic cells and regulate the gene expression in the MAPK signaling pathway.

### Expression and Transgenic Rice Plants of *PpBURP2*

To determine if *PpBURP2* responds to osmotic stress, moss collected outdoors with a 2 cm soil layer was watered with 20% PEG6000. Total RNA was extracted from gametophyte leaves for RT-qPCR. The results revealed that the expression of *PpBURP2* was induced by the PEG6000 treatment (Supplementary Figure 2A). It indicated that *PpBURP2* was involved with the response to osmotic stress. To investigate the biological function of *PpBURP2*, transgenic rice plants with over-expressed *PpBURP2* under the control of the CAMV35S promoter were produced, and T2 generation of these transgenic lines was used for further investigation. In most of the transgenic plants, *PpBURP2* could be highly expressed compared to housekeeping gene *actin1* (Supplementary Figure 2B).

### Ectopic Expression of *PpBURP2* Enhances the Tolerance to Abiotic Stress in Rice

**FIGURE 3:**
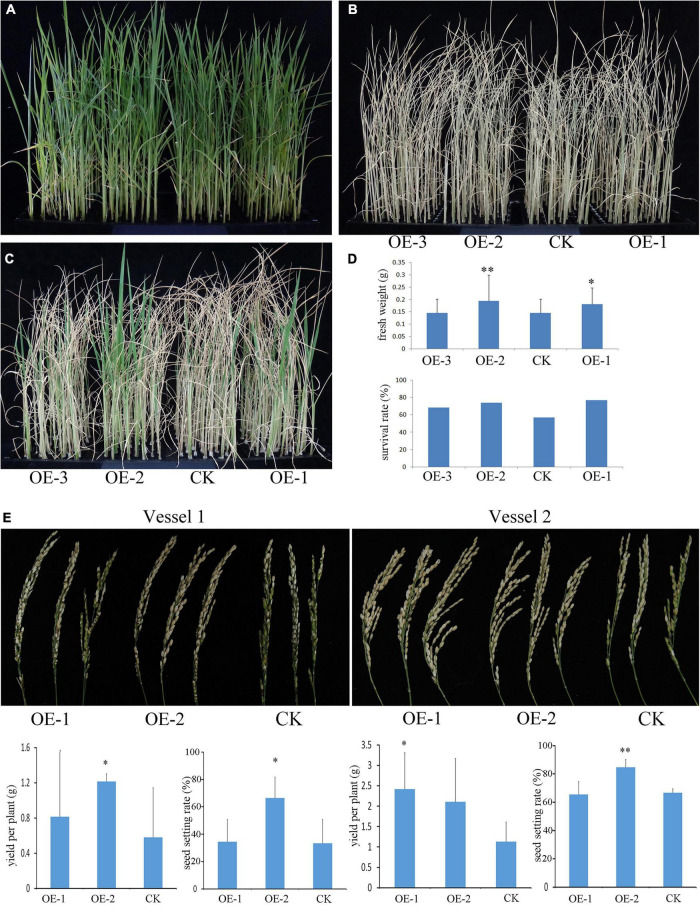
Ectopic expression of *PpBURP2* enhanced the tolerance of rice to drought stress. **(A–D)** Osmotic stress at the seedling stage. *PpBURP2* transgenic rice plants and wild-type (WT) plants were cultured in 96-well plates and treated with 18% PEG6000 for 7 days. Representative images of the WT and transgenic seedlings before **(A)**, during **(B)**, and after which they recovered for 4 days **(C)**. **(D)** Fresh weight and survival rate of *PpBURP2* transgenic plants and WT plants after PEG-simulated osmotic stress. **(E)** Drought stress at the adult stage. The transgenic plants and WT plants were grown in the same vessels with two repeats in Vessel 1 and Vessel 2 under normal conditions, respectively. At the booting stage, the water supply was withheld to cause drought stress. The upper of the images showed the panicles harvested after drought stress in Vessel 1 and Vessel 2. The yield per plant and seed setting rate in Vessel 1 and Vessel 2 were in the bottom. OE-1 and OE-2 indicate *PpBURP2* transgenic line 1 and 2. CK indicates the WT plants. The error bars indicate standard errors (SEs) based on three replicates. *p < 0.05 and ^**^p < 0.01.

In the terrestrial environment, unfavorable growth conditions often occur, such as extreme high or low temperatures, drought, high salt, and contamination of soils, by the heavy metal during plant growth and development. To evaluate the putative biological function of *PpBURP2*, we compared the growth of the transgenic rice plants and the WT plants under different abiotic stress conditions. To understand the resistance to osmotic stress at the seedling stage, 3-week-old seedlings were subjected to 18% PEG6000 for 7 days and were re-watered for 5 days. No obvious difference was found between transgenic plants and the WT under normal conditions (Figure 3A). However, as shown in Figure 3D, the survival rate of the transgenic plants was significantly higher than that of the WT under osmotic stress conditions. The analysis of fresh weight showed that a significant difference was observed between transgenic plants and WT. This result indicated that the ectopic expression of *PpBURP2* enhanced the tolerance to osmotic stress.

To evaluate the performance of the transgenic rice plants under drought stress at the adult stage, the transgenic and WT plants were grown in the same vessel under normal growth conditions until the booting stage. The water supply was withheld to cause drought stress until the leaves become completely wilted. Due to a serious decline in the number of seeds with grain filling, the panicle top in WT plants could not be hooked (Figure 3E). The seed setting rate and yield per plant of the OE-2 were significantly higher than that of WT in the two vessels. Additionally, the yield per plant of the OE-1 was significantly higher than that of WT in vessel 2 (Figure 3E). The results indicated that the ectopic expression of *PpBURP2* could improve the tolerance to drought stress at the adult stage in rice.

**FIGURE 4:**
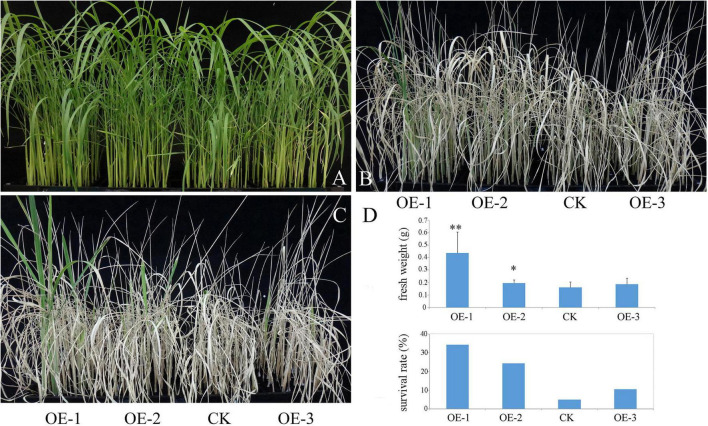
Ectopic expression of *PpBURP2* enhanced the tolerance of rice to salinity stress. *PpBURP2* transgenic rice plants and WT plants were cultured in 96-well plates and treated with 150 mM NaCl for 10 days. Representative images of the WT and transgenic seedlings before **(A)**, during **(B)**, and after which they recovered for 5 days **(C)**. **(D)** Fresh weight and survival rate of *PpBURP2* transgenic plants and WT plants after PEG-simulated osmotic stress. * *p* < 0.05 and ^**^p < 0.01.

Salinity is another brutal environmental factor limiting the productivity of crop plants. To simulate high salinity stress, 3-week-old seedlings were subjected to 150 mM NaCl for 10 days, and were re-watered for 5 days. As shown in Figure 4, the survival rate of the transgenic plants was significantly higher than that of the WT under NaCl treatment. The analysis of fresh weight showed that a significant difference was observed between transgenic plants. This result indicated that the ectopic expression of *PpBURP2* enhanced the salt tolerance.

**FIGURE 5:**
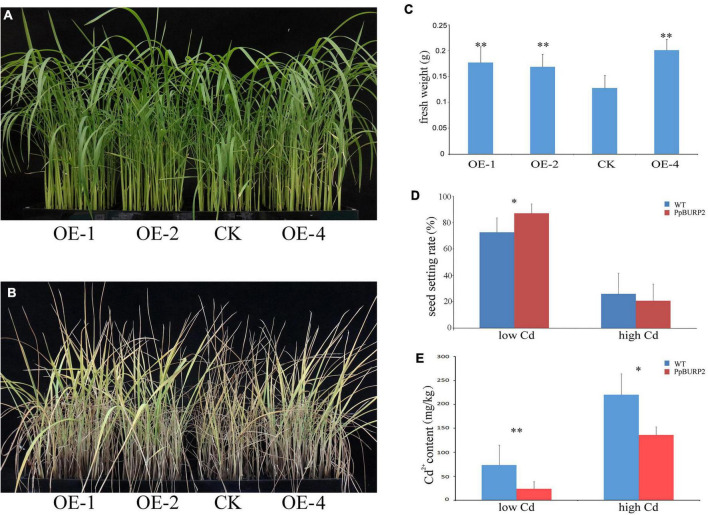
Ectopic expression of *PpBURP2* enhanced the tolerance of rice to cadmium stress. *PpBURP2* transgenic rice plants and WT plants were cultured in 96-well plates and treated with 750 mM CdCl_2_ for 10 days. Representative images of the WT and transgenic seedlings before **(A)** and after **(B)**, and fresh weight **(C)**. **(D)** Survival rate of *PpBURP2* transgenic plants and WT plants after different concentrations of CdCl_2_ treatment. **(E)** Cd^2+^ content in leaves of the WT and transgenic plants at both low and high concentration treatment. The error bars indicate SEs based on three replicates. *p < 0.05 and ^**^p < 0.01.

Rice growth is also affected by toxic concentrations of the non-essential heavy metal Cd. The growth under high Cadmium (2+) (Cd^2+^) concentration stress at the seedling stage and the adult stage was observed. At the seedling stage, no obvious difference was found between transgenic and WT plants under normal conditions (Figure 5A), but under 750 mM CdCl_2_ treatment, the leaf of WT plants firstly turned yellow (Figure 5B) and its fresh weight was significantly lower than that of the transgenic plants for 10 days (Figure 5C). At the adult stage, low concentration treatment (150 mM CdCl_2_ irrigation for 40 days) and high concentration treatment (150 mM CdCl_2_ irrigation for 80 days) were performed in the soil. The final Cd^2+^ concentration of the soil was 706.615 mg/kg in high concentration treatment and 97.035 mg/kg in low concentration treatment. The treatment of Cd^2+^ in the soil seriously affected rice tillering, especially no effective tillers in high concentration treatment. Further, low Cd^2+^ concentration treatment caused a significant decrease in the seed setting rate of WT compared to transgenic plants (Figure 5D). However, high Cd^2+^ concentration caused a sharp decrease of approximately 20% of seed setting rate in both WT and transgenic plants. The assay of Cd in leaves revealed that the Cd^2+^ content in the transgenic plants was significantly lower than in the WT in both low and high concentration treatment (Figure 5E). The results indicated that ectopic expression of *PpBURP2* improved the Cd^2+^ tolerance and reduced Cd^2+^ accumulation in rice leaves.

### Transcriptomic Analysis of Transgenic *PpBURP2* Plants

**FIGURE 6:**
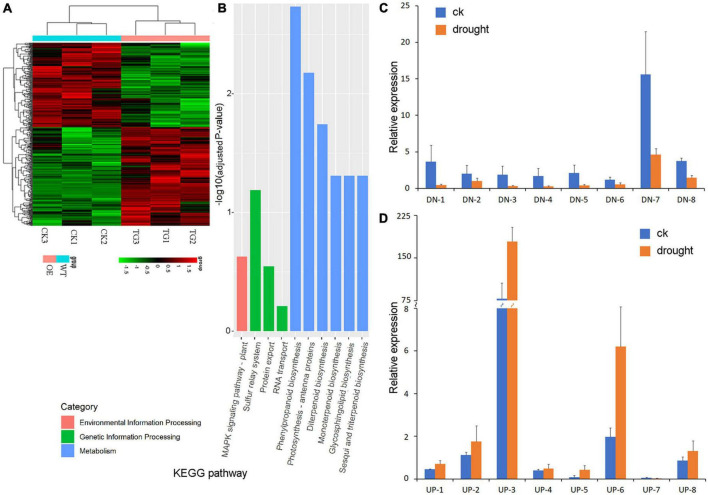
Transcriptome analysis of *PpBURP2* transgenic plants. **(A)** Clustering results of differentially expressed genes in RNA-sequencing experiment. Each row represented a log2-fold change of the relative gene expression. Upregulated expression genes were colored red, downregulated expression genes were colored green, and black represented no significant expression. CK1-3 indicates the three different samples of the WT, and TG1-3 indicates the three different samples of the PpBURP-OE1 transgenic plant. **(B)** Histogram of the Kyoto Encyclopedia of Genes and Genomes (KEGG) pathway enrichment results. The *X*-axis is the name of the pathway, and the *Y*-axis is -log10 (adjusted *p*-value) enriched by each pathway. **(C)** Relative expression level of downregulated genes in transgenic plants and WT. **(D)** Relative expression level of upregulated genes in transgenic plants and WT. The data represent the means ± SE (*n* = 3).

Ribonucleic acid-sequencing was used to analyze differentially expressed genes (DEGs) of the transgenic *PpBURP2* plants and WT. There were 179 upregulated genes (fold change ≥ 2.0) and 153 downregulated genes (fold change ≤ 0.5) in the transgenic plants compared with WT plants (Figure 6A). The expression levels of several DEGs were checked by RT-qPCR. The results confirmed the expression change of most of the selected DEGs between the transgenic and WT plants (Figures 6C,D). To understand if DEGs belong to the plant-specific genes, BlastP was performed to find DEG protein homologs in Charophytes, Chlorophyta, Bryophyta, and Animalia on the NCBI database. The results showed that 35.9% downregulated DEGs and 35.8% upregulated DEGs were found to be homologs in Charophytes, Chlorophyta, and Animalia (Supplementary Table 4). This indicated that ectopic expression of *PpBURP2*, a plant-specific gene, can regulate more than 1/3 plant-specific genes of DEGs in rice.

Gene ontology enrichment analysis revealed that the top five categories were related to membrane and ion transport in cellular component (CC) and biological process (BP), as well as terpene synthase and hexosaminidase activity in molecular function (MF). The result indicated that ectopic expression of *PpBURP2* in rice regulated metal ion homeostasis through ion transmembrane transport. Additionally, the expression of 32 genes in the oxidation-reduction process category was changed (Supplementary Table 5). Kyoto Encyclopedia for Genes and Genomes (KEGG) enrichment analysis revealed that top categories include MAPK signaling pathway in environmental information processing, protein export in genetic information processing, and photosynthesis, phenylpropanoid, and terpenoid biosynthesis in metabolism (Figure 6B and Supplementary Table 6). The result indicated that ectopic expression of *PpBURP2* in rice regulated the metabolism of secondary metabolite, energy, and oxidation-reduction process. The expression of genes related to energy metabolism is upregulated in the transgenic plants compared with WT. The expressions of eight genes involved in the phenylpropanoid, monoterpenoid, and diterpenoid biosynthesis were detected to be weakened in the transgenic plants (Supplementary Table 6). Os01g0600900 and Os04g0457000 are putative chlorophyll A/B binding proteins that are the apoproteins of the light-harvesting complex of photosystem II, and their expressions were upregulated in transgenic plants. Interestingly, DEGs are also involved in defense-related genes. *xa25* (Os12g0476200) is a dominant gene for bacterial blight resistance in rice (Chen et al., 2002), and *Pi25* (Os04g0401000) is a negative gene for the plant’s defense responses (Fukuoka et al., 2009). Additionally, NAC domain (Os01g0339500, Os03g0109000), MYB domain (Os03g0771100, Os08g0496700), and WRKY domain (Os01g0186000, Os08g0499300) contain proteins that belong to transcription factors related to abiotic and biotic stresses (Puranik et al., 2012; Jiang et al., 2017; Wang et al., 2021). The DEG data indicated that the ectopic expression of *PpBURP2* in rice altered gene expression is not only related to abiotic tolerance but are also related to biotic tolerance.

### Ectopic Expression of *PpBURP2* Affected the Photosynthesis

**FIGURE 7:**
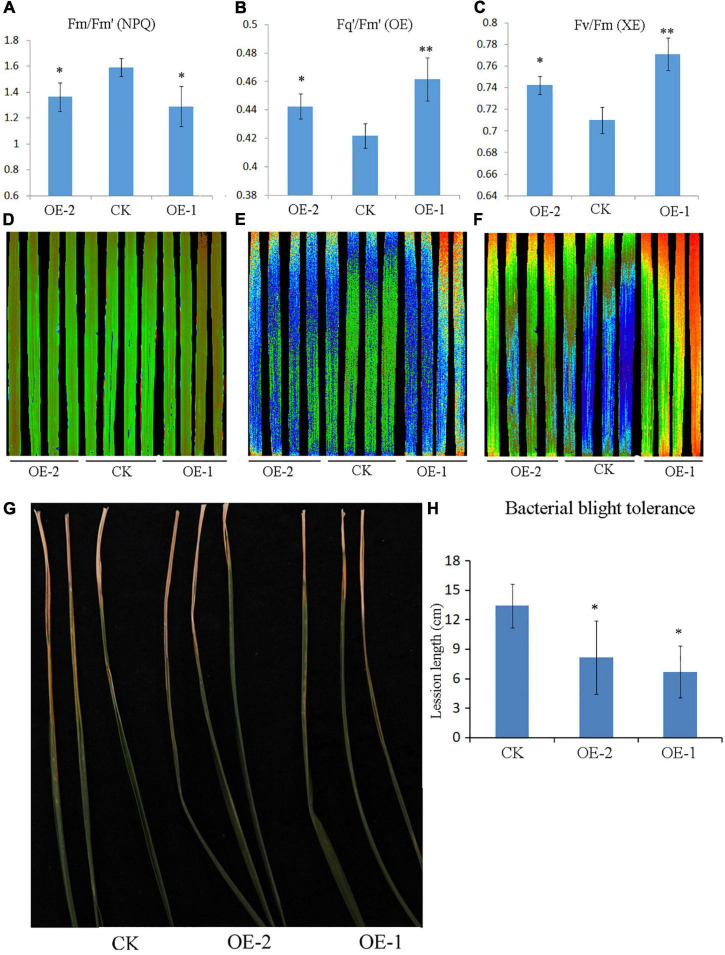
Chlorophyll fluorescence and bacterial blight resistance assay of *PpBURP2* transgenic plants. **(A–F)** Leaf photosynthetic apparatus was detected using chlorophyll fluorescence imaging. Quantification **(A)** and image **(B)** of Fm/Fm’, quantification **(B)** and image **(E)** of Fq’/Fm’, and quantification **(C)** and image **(F)** of Fv/Fm were shown at the 6th hour of measurement. **(G,H)** Inoculation of seedlings at the four-leaf stage by bacterial blight. Images **(G)** and lesion length of leaves **(H)**.

Chlorophyll fluorescence in source leaves of plants was usually used to assess the functional status of the photosynthetic apparatus. Next, we observed whether the ectopic expression of *PpBURP2* affected photosynthesis. The Fv/Fm ratio reflects the maximum capacity of absorbed quanta in photosystem II (PSII) reaction centers, and compared with WT, the Fv/Fm ratio in transgenic plants increased. Additionally, PSII operating efficiency (Fq’/Fm’) of transgenic plants was higher than WT. Non-photochemical quenching (NPQ) significantly reduced compared with WT within a day (Figures 7A–F and Supplementary Figure 3). The results suggested that the photosynthetic efficiency was obviously improved in transgenic plants.

### Ectopic Expression of *PpBURP2* Enhanced the Resistance to Bacterial Blight

To test whether PpBURP2 might have a role in disease resistance, the transgenic plants were inoculated by *Xoo*. The lesion length in two transgenic plants was considerably shorter than in WT plants (Figures 7G,H). The results indicated that the ectopic expression of *PpBURP2* showed stronger resistance against bacterial leaf blight.

### Ectopic Expression of *PpBURP2* Affected the Redox Equilibrium

**FIGURE 8:**
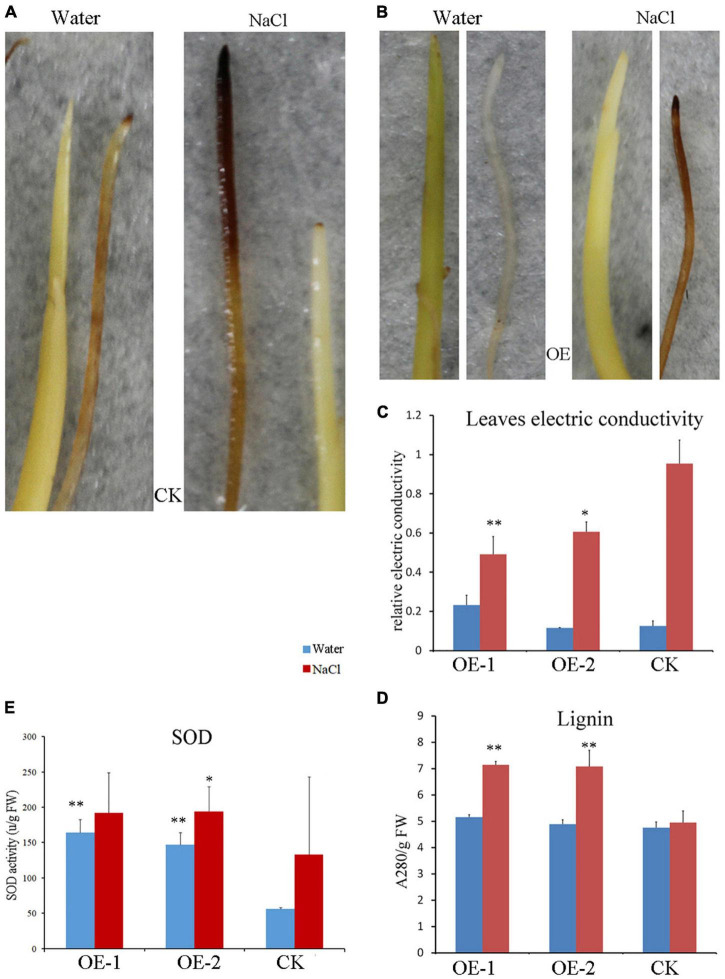
Reactive oxygen species (ROS) level and lignin assay of *PpBURP2* transgenic plants. **(A)** Images of the WT (CK) root and bud infiltrated with DAB under water and 100 mM NaCl treatments during seed germination. **(B)** Images of *PpBURP2*-OE1 (OE) root and bud infiltrated with 3,3′-diaminobenzidine (DAB) under water and 100 mM NaCl treatments during seed germination. **(C)** Leaf electric conductivity after 100 mM NaCl and water treatments. **(D)** Lignin content in leaf after 100 mM NaCl and water treatments. **(E)** Superoxide dismutase (SOD) activity in leaf after 100 mM NaCl and water treatments. OE-1 and OE-2 indicate transgenic *PpBURP2* lines 1 and 2, and CK indicates the WT. The error bars indicate SEs based on three replicates. The *t*-test was performed between OE and CK in same color columns. *p < 0.05 and ^**^p <0.01.

To detect reactive oxygen species (ROS), the 3,3′-diaminobenzidine (DAB) staining methods were used in rice seedlings. The images showed that the rice root and shoot of the WT line were colored under normal conditions but were not of the transgenic lines (Figures 8A,B). Further, there was a significant difference in color on the root between WT and the transgenic plant under salt stress, in which the color became more intense overall in WT plants (Figures 8A,B). The results indicated that overexpressing *PpBURP2* lines can produce less ROS than WT. Whereafter, SOD levels are used to represent the redox equilibrium of cells under stresses. The relative levels of SOD were significantly elevated. The SOD activity in OE lines was higher than the WT with or without NaCl treatment (Figure 8E). In contrast, leaf electric conductivity was elevated after NaCl treatment, but electric conductivity of transgenic lines was significantly lower than that of WT (Figure 8C). Additionally, lignin content was elevated in leaf after NaCl treatment and the lignin content of transgenic lines was significantly higher than that of WT (Figure 8D). It indicated that ectopic expression of *PpBURP2* could enhance the SOD activity and lignin content, and reduce the accumulation of ROS and damage of cell membrane after salt stress.

## Discussion

To thrive on land, plants require sturdy cell walls that enable an erect body plan and protection against abiotic stresses, such as ultraviolet radiation, drought, and varying temperature (Rensing, 2018). As the first shelter, the structure and composition of the cell wall and cell periphery are complex. For moss, adaptation to land required the evolution of proteins that protect against abiotic stresses. BURP gene family had occurred in the genomes of Bryophyta (Supplementary Figure 1). In addition, PpBURP2 protein was found to be localized to the apoplast (Figure 2A). BURP proteins in higher plants were also localized to the cell wall and apoplast (Wang et al., 2012; Xu et al., 2013; Park et al., 2015). In cotton, GhRDL1 interacted with GhEXPA1 to loosen the cell wall and promote cell enlargement (Xu et al., 2013). Similar to other higher plants, the expression of *PpBURP2* was induced by osmotic stress in moss (Supplementary Figure 2A). Ectopic expression of *PpBURP2* in rice enhanced the tolerance to drought, salinity, high Cd^2+^, and bacterial blight (Figures 3–5). Moreover, one-third of DEGs regulated by *PpBURP2* in rice belong to plant-specific genes, indicating that the expression net constructed by plant-specific genes is an essential foundation of adaption to the land environment of plants (Supplementary Table 4). Our results indicate that BURP proteins in early land plants evolved into an important component of cell adaptation to land through participating in the development of cell wall and responding to abiotic and biotic stresses.

Since the denomination of BURP-domain, this protein was reported to be mainly involved in seed, anther development, and abiotic stress. The plant-specific BURP-containing protein family is critical in the adaptation of plants to adverse environments. Analysis of plant-specific genes in DEGs revealed that ectopic expression of *PpBURP2* could regulate many plant-specific genes to adapt to land environment in rice (Supplementary Table 3). To know how to adapt to an adverse environment, protein-protein interaction information is an important channel. GmRD22 interaction with a putative apoplastic peroxidase was thought to enhance lignin content (Wang et al., 2012). Another result revealed that GhRDL1 interacted with expansin and promoted plant development (Xu et al., 2013). In this study, OsMKK1 was verified to interact with BURP proteins from moss and rice. Plant MAPK cascades are prevalent in eukaryote to play a key role in biotic and abiotic stress responses, hormone responses, cell division, and development (Yu and Tang, 2004). For example, OsMKK1 and OsMPK4 were found to constitute a signaling pathway that regulates salt stress tolerance in rice (Wang et al., 2014), so much so that they regulate crosstalk between abiotic and biotic stress (Yoo et al., 2014). MKK10.2 promotes disease resistance and drought tolerance by activating different MAPKs in rice (Ma et al., 2017). Therefore, we infer that BURP proteins are involved in stress signal transduction in a conserved way through the protein-protein interaction with MKK proteins in the plant kingdom. Apoplast localization of a single BURP protein suggests that it may play an unknown role in signaling on environmental stresses. MKK proteins were localized to the cytoplasm or trafficked to the nucleus (Liang et al., 2013). Co-localization of OsMKK1 and the two BURP proteins indicated that BURP proteins could be trafficked to the nucleus with MKK proteins. In Arabidopsis, a MAPK kinase (MEKK2) is a scaffolding protein without kinase activity (Liu et al., 2020). The BURP proteins may be used as a scaffold, phosphorylated target, or activator with the interaction of MKK. However, the role of the BURP proteins needs to be further investigated.

**FIGURE 9:**
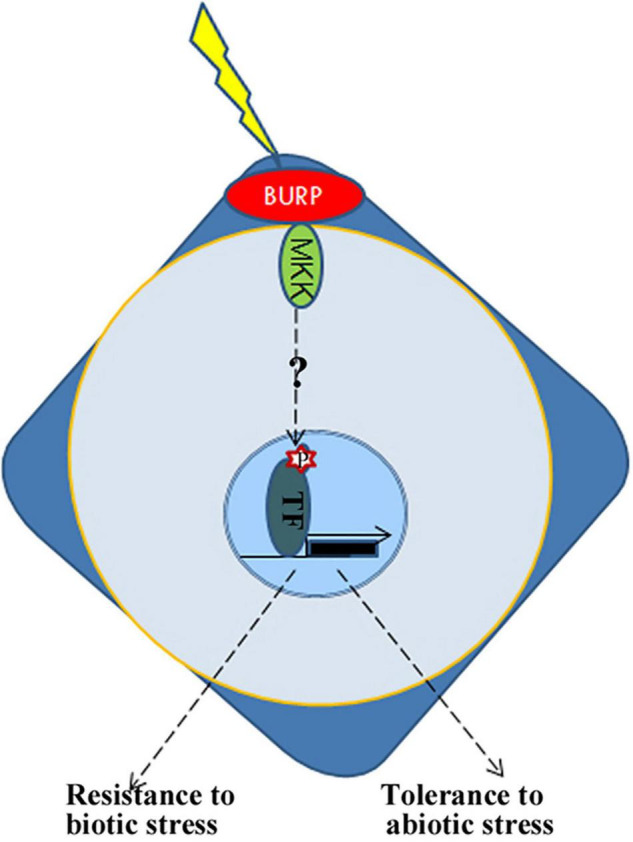
Schematic representation of possible roles for BURPs in integrating abiotic and biotic stress responses. BURP, BURP-containing protein; MKK, Mitogen-activated protein kinase kinase; TF, transcription factor; P, phosphorylation.

From the above results, it is inferred that BURP protein is an important member of the plant signaling pathway. Transcriptomic analysis based on RNA-sequencing also revealed that the ectopic expression of *PpBURP2* in rice could regulate biotic and abiotic stress responses, biological metabolism, and development. The process included metal ion homeostasis, oxidation-reduction process, MAPK signaling, secondary metabolite, and energy process. On the one hand, PpBURP2 was participated in abiotic stress response. Phenotypically, we observed that the transgenic *PpBURP2* plants were more resistant to drought, salinity, and heavy metal. Meanwhile, the gene expression of chlorophyll A/B binding proteins was elevated, and the photosynthetic efficiency was improved in the transgenic plants (Figures 7A–F). Finally, ectopic expression of *PpBURP2* in rice could enhance the tolerance to abiotic stress and increase production under abiotic stress (Figures 3–5). On the other hand, PpBURP2 was involved in biotic defense response. The improvement of disease resistance of *PpBURP2-*overexpressed rice plants is due to the altered expression of the defense-related gene *xa25* and *Pi25* (Figure 7G). The regulation of MAPK signaling and the interaction between PpBURP2 and OsMKK1 are thought to be a way for BURP proteins to participate in stress response signal transduction in plants. WRKY and MYB transcription factors have been identified as substrates of MAPK cascades involved in innate immune response and abiotic stresses (Colcombet and Hirt, 2008; Kim et al., 2017). NAC transcription factors are key components in transcriptional regulation of gene expression during pathogen attack and abiotic stresses (Puranik et al., 2012). Phosphorylation of a NAC transcription factor can enhance the drought tolerance in maize (Han et al., 2021). In DEGs, there are two WRKY genes [*WRKY10* (*Os01g0186000*) and *WRKY30* (*Os08g0499300*)], two NAC genes [*NAC30* (*Os01g0339500*), *NAC55* (*Os03g0109000*)], and one *MYB* gene (*Os03g0771100*). Moreover, these DEG-encoded proteins are potential phosphorylated targets of MAPK cascades. In higher plants, the BURP family has been shown to be involved in plant development and stress response (Wang et al., 2015; Li et al., 2016; Sun et al., 2019). We propose a hypothesis that the BURP-MKK signal pathway is a conserved pathway to mediate response to biotic and abiotic stress in the plant kingdom (Figure 9). However, details on the signal transduction needs more investigation. In a word, as a member of BURP domain family, PpBURP2 plays an important role in the tolerance to multiple abiotic and biotic stresses.

## Data Availability Statement

The raw Illumina sequencing data used in this study are available in the NCBI Sequence Read Archive (https://www.ncbi.nlm.nih.gov/sra/) under the accession number PRJNA754445.

## Author Contributions

SY designed the experiments, performed subcellular localization, stress treatments, physiological analysis, and wrote the manuscript. FY, YZ, YW, and YY carried out the yeast assay, qPCR, plant culture, biochemical assay, and vector construction. TL performed the transformation of rice. SC and KX carried out the gene expression analysis and the data analysis. HX and LL supervised this work and assisted with editing the manuscript. All authors read and approved the final manuscript.

## Conflict of Interest

The authors declare that the research was conducted in the absence of any commercial or financial relationships that could be construed as a potential conflict of interest.

## Publisher’s Note

All claims expressed in this article are solely those of the authors and do not necessarily represent those of their affiliated organizations, or those of the publisher, the editors and the reviewers. Any product that may be evaluated in this article, or claim that may be made by its manufacturer, is not guaranteed or endorsed by the publisher.

## Footnotes

1
https://plants.ensembl.org/Physcomitrium_patens

2
http://genome.jgi-psf.org/

3
http://rice.plantbiology.msu.edu

4
http://www.arabidopsis.org

5
https://www.ebi.ac.uk/Tools/pfa/radar/

6
https://www.ncbi.nlm.nih.gov/

7
http://geneontology.org/

8
http://rice.uga.edu/
