# Supplementary material for: Overexpressing PpBURP2 in Rice Increases Plant Defense to Abiotic Stress and Bacterial Leaf Blight
Source: Front Plant Sci. 2022 May 6;13:812279. doi: 10.3389/fpls.2022.812279 (PMC9121072; doi:10.3389/fpls.2022.812279)

Supplementary information for

PpBURP2 play an important role in the conquest of land by plants

Shunwu Yu, Fangwen Yang, Yunan Yang, Yuqiao Zou, Tianfei Li, Shoujun Chen, Kai Xu, Lijun Luo

Supplementary Figure 1. Intron–exon organization of BURP family.

Supplementary Figure 2. The relative expression level of *PpBURP2* gene in moss and the transgenic rice.

Supplementary Figure 3. Leaf photosynthetic quantification was detected using chlorophyll fluorescence imaging for every hour per cycle 24 cycles.

Supplementary Table 1. Gene lists of moss and spikemoss

Supplementary Table 2. Motif structure number of BURP domain-containing proteins from diverse species

Supplementary Table 3. BlastP of DEGs

Supplementary Table 4.Lists of GO pathway enrichment of differentially expressed genes between *PpBURP2* transgenic plant and WT.

Supplementary Table 5. Lists of KEGG pathway enrichment of differentially expressed genes between *PpBURP2* transgenic plant and WT.

Supplementary Table 6. Putative interaction protein of OsBURP3 from RicePPINet with the value over 0.8 in rice.

Supplementary Table 7. Primers used for qPCR

Supplementary Figure 1. Intron–exon organization of BURP family.


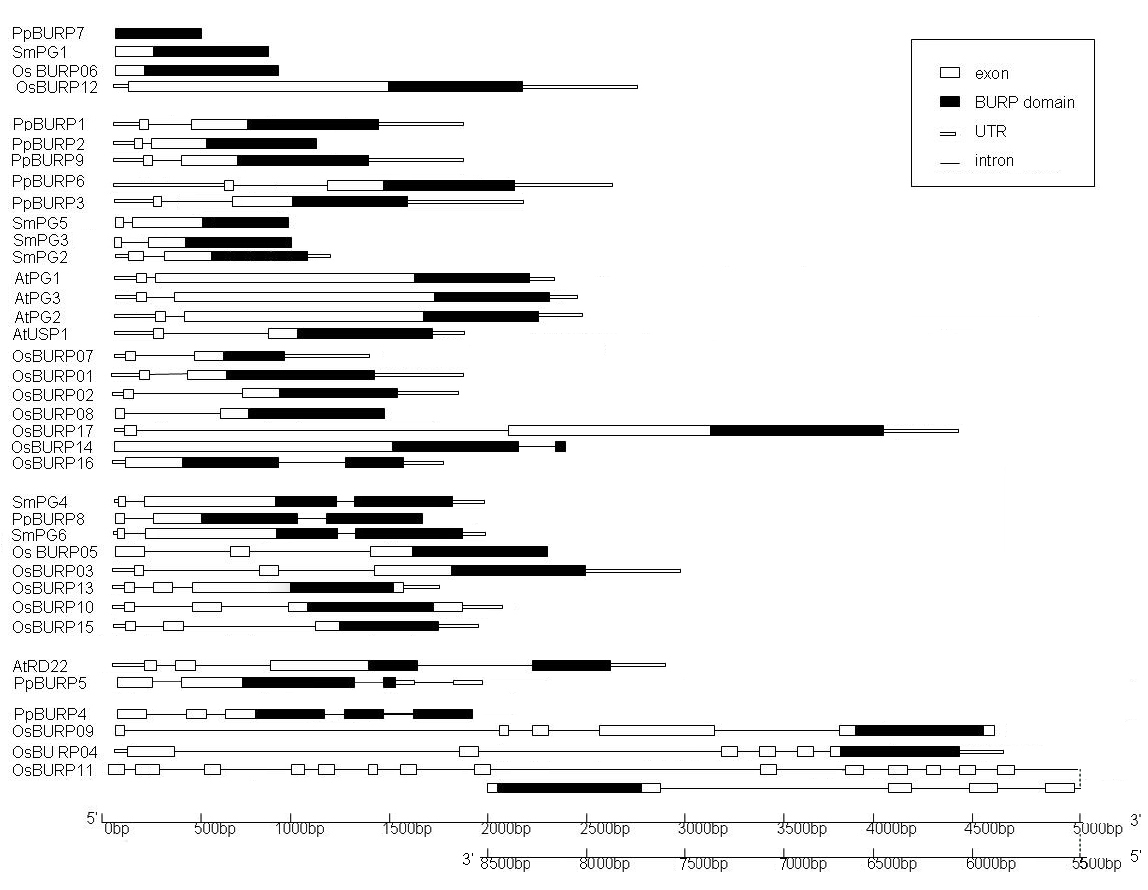


White rectangles and thin lines represent exons and introns, respectively. Thick lines represent untranslated regions. Black rectangles represent the BURP domains. For based on MSU Rice Genome Annotation Project Release 7, intron-exon organization of the three *OsBURP* genes is different from Ding’s report, such as *OsBURP07* without a intron in BURP domain, *OsBURP17* with only one intron and *OsBURP11* with more ten exons in 3’ end.

Supplementary Figure 2. The relative expression level of *PpBURP2* gene in moss and the transgenic rice. (A) Gametophyte leaves of moss 20% PEG6000 treatment, (B) leaves of *PpBURP2* transgenic plants.


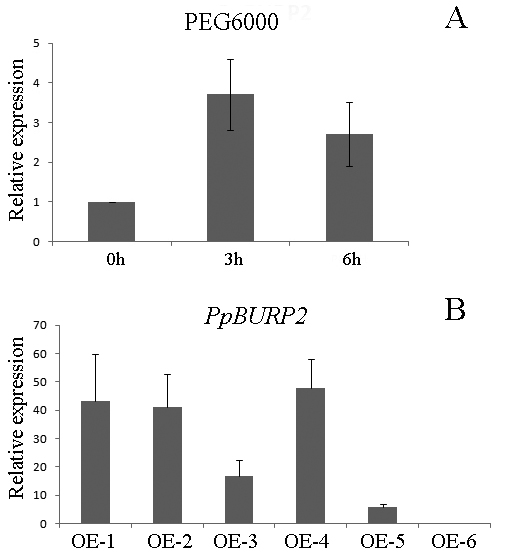


Supplementary Figure 3. Leaf photosynthetic quantification was detected using chlorophyll fluorescence imaging for every hour per cycle 24 cycles.


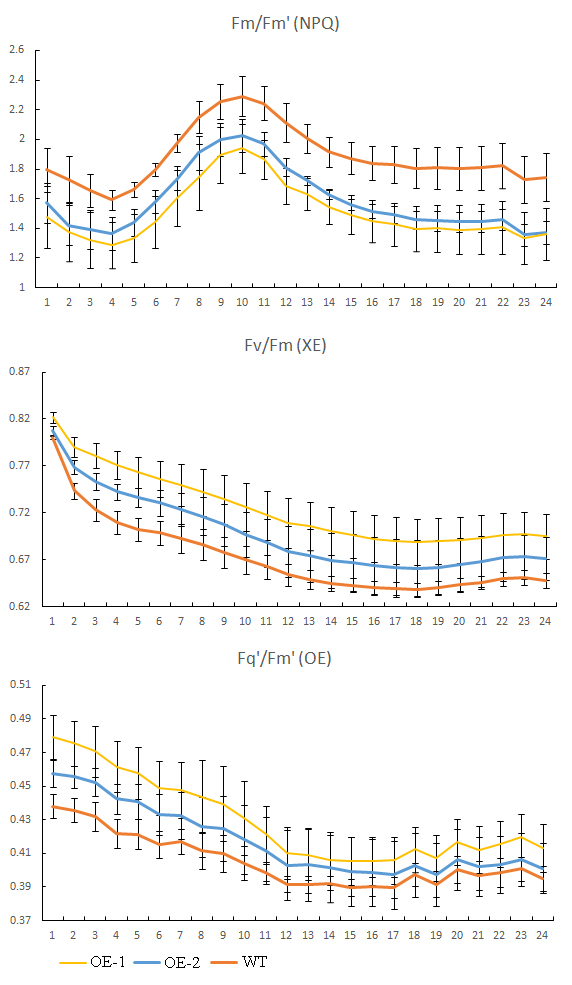

Supplement: Supplementary file 1 [file Data_Sheet_1.docx]
