# Supplementary material for: Overexpressing PpBURP2 in Rice Increases Plant Defense to Abiotic Stress and Bacterial Leaf Blight
Source: Front Plant Sci. 2022 May 6;13:812279. doi: 10.3389/fpls.2022.812279 (PMC9121072; doi:10.3389/fpls.2022.812279)
Supplement: Supplementary file 2 [file Table_1.docx]

| organisms | gene | Sequenc ID | AA length | PI |
| --- | --- | --- | --- | --- |
| *Physcomitrella patens* | PpBURP1 | Pp1s204_70V6.1 | 348 | 5.91 |
|  | PpBURP2 | Pp1s374_47V6.1 | 350 | 8.72 |
|  | PpBURP3 | Pp1s298_14V6.1 | 342 | 5.93 |
|  | PpBURP4 | Pp1s81_232V6.1 | 396 | 5.83 |
|  | PpBURP5 | Pp1s7_236V6.1 | 340 | 5.95 |
|  | PpBURP6 | Pp1s56_133V6.1 | 337 | 4.99 |
|  | PpBURP7 | Pp1s26_27V6.1 | 190 | 5.90 |
|  | PpBURP8 | Pp1s281_126V6.1 | 413 | 5.02 |
|  | PpBURP9 | Pp1s15_23V6.1 | 391 | 5.50 |
| *Selaginella moellendorffii* | SmPG1 | XP_024528164.1 | 285 | 6.08 |
|  | SmPG2 | XP_024536418 | 294 | 6.98 |
|  | SmPG3 | XP_002962118.2 | 304 | 8.86 |
|  | SmPG4 | XP_024515919.1 | 597 | 7.00 |
|  | SmPG5 | XP_024538407 | 283 | 8.63 |
|  | SmPG6 | XP_024514723.1 | 641 | 6.84 |

Supplementary Table 1 Basic information of the BURP proteins in moss and spikemoss
